# Patient-reported outcomes and survival in multiple sclerosis: A 10-year retrospective cohort study using the Multiple Sclerosis Impact Scale–29

**DOI:** 10.1371/journal.pmed.1002346

**Published:** 2017-07-10

**Authors:** Joel Raffel, Alison Wallace, Djordje Gveric, Richard Reynolds, Tim Friede, Richard Nicholas

**Affiliations:** 1 Division of Brain Sciences, Faculty of Medicine, Imperial College London, London, United Kingdom; 2 Imperial College Healthcare NHS Trust, London, United Kingdom; 3 Department of Medical Statistics, University Medical Center Göttingen, Göttingen, Germany; Stanford University, UNITED STATES

## Abstract

**Background:**

There is increasing emphasis on using patient-reported outcomes (PROs) to complement traditional clinical outcomes in medical research, including in multiple sclerosis (MS). Research, particularly in oncology and heart failure, has shown that PROs can be prognostic of hard clinical endpoints such as survival time (time from study entry until death). However, unlike in oncology or cardiology, it is unknown whether PROs are associated with survival time in neurological diseases. The Multiple Sclerosis Impact Scale–29 (MSIS-29) is a PRO sensitive to short-term change in MS, with questions covering both physical and psychological quality of life. This study aimed to investigate whether MSIS-29 scores can be prognostic for survival time in MS, using a large observational cohort of people with MS.

**Methods and findings:**

From 15 July 2004 onwards, MSIS-29 questionnaires were completed by people with MS registered with the MS Society Tissue Bank (*n* = 2,126, repeated 1 year later with *n* = 872 of the original respondents). By 2014, 264 participants (12.4%) had died. Higher baseline MSIS-29 physical (MSIS-29-PHYS) score was associated with reduced survival time (subgroup with highest scores versus subgroup with lowest scores: hazard ratio [HR] 5.7, 95% CI 3.1–10.5, *p <* 0.001). Higher baseline MSIS-29 psychological score was also associated with reduced survival time (subgroup with highest scores versus subgroup with lowest scores: HR 2.8, 95% CI 1.8–4.4, *p <* 0.001). In those with high baseline MSIS-29 scores, mortality risk was even greater if the MSIS-29 score worsened over 1 year (HR 2.3, 95% CI 1.2–4.4, *p* = 0.02). MSIS-29-PHYS scores were associated with survival time independent of age, sex, and patient-reported Expanded Disability Status Scale score in a Cox regression analysis (per 1-SD increase in MSIS-29-PHYS score: HR 1.8, 95% CI 1.1–2.9, *p* = 0.03). A limitation of the study is that this cohort had high baseline age and disability levels; the prognostic value of MSIS-29 for survival time at earlier disease stages requires further investigation.

**Conclusions:**

This study reports that PROs can be prognostic for hard clinical outcomes in neurological disease, and supports PROs as a meaningful clinical outcome for use in research and clinical settings.

## Abstract 2

In a retrospective cohort study of 2,126 patients with multiple sclerosis, Joel Raffel of Imperial College London and colleagues showed that the scores from a patient-reported outcomes questionnaire were associated with the patients' 10-year risk of mortality.

## Author summary

**Why was this study done?:**

- Patient-reported outcomes (PROs) are questionnaires completed by people with a given condition, to help capture the impact of disease or treatment on the individual.
- PROs have many uses, e.g., to screen for symptoms, assess treatment response, and enhance doctor–patient communication.
- However, they are still underused in neurological conditions, in part because it is not clear if PROs relate to ‘hard clinical outcomes’ like disability or mortality.
- The Multiple Sclerosis Impact Scale–29 (MSIS-29) questionnaire is a PRO that assesses quality of life in people with multiple sclerosis (MS).
- Our study aimed to investigate whether MSIS-29 score is associated with the future risk of death in a large cohort of people with MS.

**What did the researchers do and find?:**

- MSIS-29 questionnaires were completed by 2,126 people with MS in the UK. The questionnaire was repeated 1 year later in 872 people.
- Of the 2,126 participants, 264 died over 10 years of follow-up.
- We found that the MSIS-29 scores of participants were associated with their risk of 10-year mortality, even after adjusting for known risk factors for mortality such as age, sex, and baseline disability score.
- The subgroup with the highest MSIS-29 physical scores (indicating the poorest physical quality of life) had a 5.7 times greater risk of death than the subgroup with the lowest scores. Mortality risk was even higher in those for whom the MSIS-29 score worsened over 1 year.

**What do these findings mean?:**

- To our knowledge, this is the first study to associate PROs with risk of mortality in neurological disease.
- This study shows that how a person with MS answers the MSIS-29 questions is important and relates to how well they may do in terms of health in the future.
- A limitation of the study is that participants with early disease and minimal disability were underrepresented.
- To improve neurological care, we must incorporate PROs into automated electronic systems to serve both routine clinical care and large-scale data collection. This will allow us to address key issues such as comparing the effectiveness of treatment options and tailoring treatment options to an individual’s needs.

## Introduction

Patient-reported outcomes (PROs) are defined as ‘any report of the status of a patient’s health condition that comes directly from the patient, without interpretation of the patient’s response by a clinician or anyone else’ [1]. They can offer significant advantages over assessment by a physician: they better capture the impact of disease on the person; they are often easier and cheaper to administer; and they can often be completed from the home environment, potentially allowing for long-term, geographically diverse, and large-scale observational and interventional studies [2]. They can also enhance routine clinical care in areas such as symptom screening, monitoring treatment response, care coordination, care systems assessment, and improving communication in the doctor–patient clinical encounter [3–6].

PROs are increasingly being used to complement traditional outcome measures in disciplines such as oncology, cardiology, and neurology. The increasing use of PROs in interventional trials is partly driven by the need for pharmaceutical companies to justify labelling and promotional claims in post-licensing marketing [1,7]. However, research and clinical practice, particularly in oncology, have led the way in proving that PROs can offer more than this: it is now well established in oncology that PROs are associated with hard clinical endpoints such as survival time (time from study entry until death) and can add prognostic value to the more traditional physician-reported outcome measures [8–10]. PROs are also well established as prognostic for survival time in heart failure [11–13]. However, such associations are more difficult to study in neurological research, in part because it is rarer for the clinical endpoint of trials in neurological disease to be survival.

The Multiple Sclerosis Impact Scale–29 (MSIS-29) is a PRO that attempts to assess both physical and psychological quality of life in multiple sclerosis (MS) [14]. It has the advantage of being self-reported and can be distributed by post. It could potentially be utilised in progressive MS trials, as it appears sensitive to clinically relevant change over short time frames [15]. This is in contrast to the traditional physician-assessed Expanded Disability Status Scale (EDSS), which is the primary outcome favoured in most MS trials despite several well-documented limitations, including poor interrater and intrarater reliability and a limited sensitivity to change over the time frame of 2–3 years, especially in progressive MS [16–20]. Though small studies have correlated MSIS-29 score with EDSS score, it is unknown how the MSIS-29 is linked to robust clinical endpoints such as survival time [21,22]. Indeed, to our knowledge, no PROs have been associated with survival in MS or any other neurological disease.

This study aimed to investigate whether MSIS-29 scores can be prognostic for survival time (time from MSIS-29 completion to death) in MS, using a large observational cohort of people with MS from the MS Society Tissue Bank (MSSTB). The primary study hypothesis was that MSIS-29 scores are associated with survival time in MS.

## Methods

### Study population

Since 1998, the MSSTB has operated a nationwide community-based scheme for people with MS and non-MS controls in the UK to donate their brain and spinal cord after death, by providing written consent while alive (ethics approval in 1998: London Multicentre Research Ethics Committee—MREC/02/2/39; then in 2008: Wales Research Ethics Committee 3–08/MRE09/31; then in 2013: Wales Research Ethics Committee 3–08/MRE09/31+5) [23]. This cohort is a unique population in that the participants are followed from registration to death, with eventual pathological confirmation [23]. On 15 July 2004, MSIS-29 questionnaires were sent out to all registered donors. For those who completed a MSIS-29 questionnaire at this time, a second MSIS-29 questionnaire was sent out 1 year later, to measure change in MSIS-29. In addition, since 15 July 2004, all new registered donors have been sent a MSIS-29 questionnaire at the time of registration, as well as a ‘patient-reported EDSS’ (prEDSS) [24,25]. This study included people with MS registered in the MSSTB up until 1 January 2014 who had completed at least 1 MSIS-29 questionnaire. Data were stored in the MSSTB facility at Imperial College London and were analysed in 2015–2016.

### Outcome measures

The MSIS-29 consists of 29 questions answered on a 5-point Likert scale, giving 2 scores: the MSIS-29 physical (MSIS-29-PHYS) score (questions 1–20; therefore score range 20–100) and the MSIS-29 psychological (MSIS-29-PSYCH) score (questions 21–29; therefore score range 9–45) [14,26]. Imputation was used to address questionnaires returned with missing data using the following rule: if greater than 66% of questions had been answered within MSIS-29-PHYS or within MSIS-29-PSYCH, missing answers were imputed using the mean of the answered questions from the MSIS-29-PHYS or MSIS-29-PSYCH of that individual participant [27]. Otherwise, all data from that MSIS-29-PHYS or MSIS-29-PSYCH were excluded. The EDSS is a physician-reported gold standard for categorising disability in MS. The minimum score of 0 represents no impairment, 7 represents restriction to wheelchair, and the maximum score of 10 represents death. The prEDSS uses the same scale and can be completed without physician input, with good correlation [24]. A description of how to request access to the MSIS-29 questionnaire and the prEDSS questionnaire is available in S1 Appendix. Ten-year data on mortality were collected up until 1 June 2014. Survival time was defined using date of first MSIS-29 questionnaire as the entry point, date of death as the endpoint, and date of study completion (1 June 2014) as the censorship date for those still alive.

Baseline MSIS-29 scores were categorised into 5 equally spaced subgroups as follows: MSIS-29-PHYS scores—20–35, 36–51, 52–68, 69–84, 85–100; MSIS-29-PSYCH scores—9–16, 17–23, 24–30, 31–37, 38–45. In addition, for those with a repeat MSIS-29 questionnaire 1 year after baseline, the following subgroups were used: Subgroup 1—initial MSIS-29-PHYS score 20–84, no worsening after 1 year; Subgroup 2—initial MSIS-29-PHYS score 20–84, worsening after 1 year (≥1 point); Subgroup 3—initial MSIS-29-PHYS score 85–100, no worsening after 1 year; and Subgroup 4—initial MSIS-29-PHYS score 85–100, worsening after 1 year (≥1 point).

### Statistical analysis

We did not preregister or publish a detailed analysis plan for this study. The statistical analysis is described below. Details on the history of this study and changes to the analysis plan are provided in S2 Appendix, while an early project outline is provided in S3 Appendix.

Population demographics are presented as mean (standard deviation [SD]) and frequency (percentage) for continuous and categorical variables, respectively. Differences between continuous variables were tested with the unpaired Student's *t*-test, or one-way analysis of variance (ANOVA) when more than 2 groups, while differences between categorical data were tested with the chi-square test. To assess whether MSIS-29-PHYS score, MSIS-29-PSYCH score, prEDSS score, and change in MSIS-29-PHYS score are associated with mortality, survival times were modelled using Cox proportional hazard models where the hazard ratios (HRs) for the respective instruments were adjusted for age and sex. The HRs are presented with 95% confidence intervals (95% CIs) and *p*-values testing the null hypothesis of the HRs being equal to 1. Survival curves within the subgroups were estimated using Kaplan–Meier estimators. Correlations between the 3 PRO scales (MSIS-29-PHYS, MSIS-29-PSYCH, and prEDSS) were estimated using rank-based Spearman correlations, which are reported with *p*-values testing the null hypothesis of no correlation. To investigate whether MSIS-29-PHYS and MSIS-29-PSYCH scores are associated with survival independent of prEDSS score, survival times were modelled using a Cox regression with prEDSS score, MSIS-29-PHYS score, MSIS-29-PSYCH score, age, and sex as independent variables. The SAS 9.4 platform was used for all statistical analysis.

## Results

### Population demographics

In all, 2,914 people with MS were enrolled in the MSSTB over the study period. 2,126 participants completed the MSIS-29 questionnaire for inclusion in this study (participation rate 73.0%). Of these, 2,119 participants completed both the MSIS-29-PHYS and MSIS-29-PSYCH questionnaire, and 7 participants completed only the MSIS-29-PHYS questionnaire. Data were imputed for 273 participants. A prEDSS assessment was available at the same time as the MSIS-29 assessment in 630 participants, and a repeat MSIS-29 questionnaire was completed 1 year after baseline by 872 participants. Differences in baseline characteristics between those included and those not included in the study, those with and without imputed data, those with and without prEDSS data, and those with and without longitudinal MSIS-29 data are presented in Table 1.

**Table 1:** Differences in baseline characteristics between those included and those not included in the study, those with and without imputed data, those with and without prEDSS data, and those with and without longitudinal MSIS-29 data.

Characteristic	*n*	Male sex	Age	Disease duration	Baseline MSIS-29-PHYS	Baseline MSIS-29-PSYCH
**Included in the study**						
Yes	2,126	496 (23.3%)	54.0 (11.9)	18.5 (15.0)	62.0 (20.5)	23.6 (8.7)
No	788	182 (23.1%)	55.8 (13.3)	18.4 (11.3)	n/a	n/a
*p-*Value		0.894	**<0.001**	0.827		
**Imputed data**						
No	1,853	439 (23.7%)	53.5 (11.7)	17.9 (14.5)	61.4 (20.5)	23.6 (8.8)
Yes	273	57 (20.9%)	57.4 (12.9)	23.3 (16.8)	65.8 (20.4)	23.3 (8.6)
*p-*Value		0.305	**<0.001**	**<0.001**	**0.001**	0.484
**prEDSS completed**						
Yes	630	152 (24.1%)	50.9 (12.3)	16.9 (17.0)	62.1 (20.4)	23.8 (8.7)
No	1,496	344 (23.0%)	55.2 (11.5)	19.2 (13.9)	61.9 (20.6)	23.5 (8.7)
*p-*Value		0.573	**<0.001**	**<0.001**	0.909	0.457
**1-year repeat MSIS-29 completed**						
Yes	872	188 (21.6%)	55.1 (11.1)	18.2 (13.4)	60.6 (19.5)	23.0 (8.3)
No	1,254	308 (24.6%)	53.1 (12.4)	18.8 (15.9)	63.0 (21.1)	24.0 (9.0)
*p-*Value		0.107	**<0.001**	0.407	**0.008**	**0.007**

Data are presented as n (percent) or mean (SD). Differences between categorical data (sex) were tested with the chi-square test. Differences between continuous data (age, disease duration, MSIS-29-PHYS, MSIS-29-PSYCH) were tested with the unpaired Student's t-test. Significant p-values are highlighted in bold.

MSIS-29, Multiple Sclerosis Impact Scale–29; MSIS-29-PHYS, MSIS-29 physical; MSIS-29-PSYCH, MSIS-29 psychological; n/a, not applicable; prEDSS, patient-reported Expanded Disability Status Scale.

Median follow-up time was 9 years, while 865 participants had the maximum 10 years of follow-up. Up until 1 June 2014, 264 (12.4%) of the total group had died. The mean population age at MSIS-29 assessment was 54 (SD 11.9) years, with a disease length of 18.5 (SD 15.0) years, and 1,630 (76.7%) were female. At baseline, mean MSIS-29-PHYS score was 62 (SD 20.5), mean MSIS-29-PSYCH score was 23.6 (SD 8.7), and mean prEDSS score was 5.7 (SD 2.2).

### A higher MSIS-29-PHYS score is associated with reduced survival time

**Fig 1:**
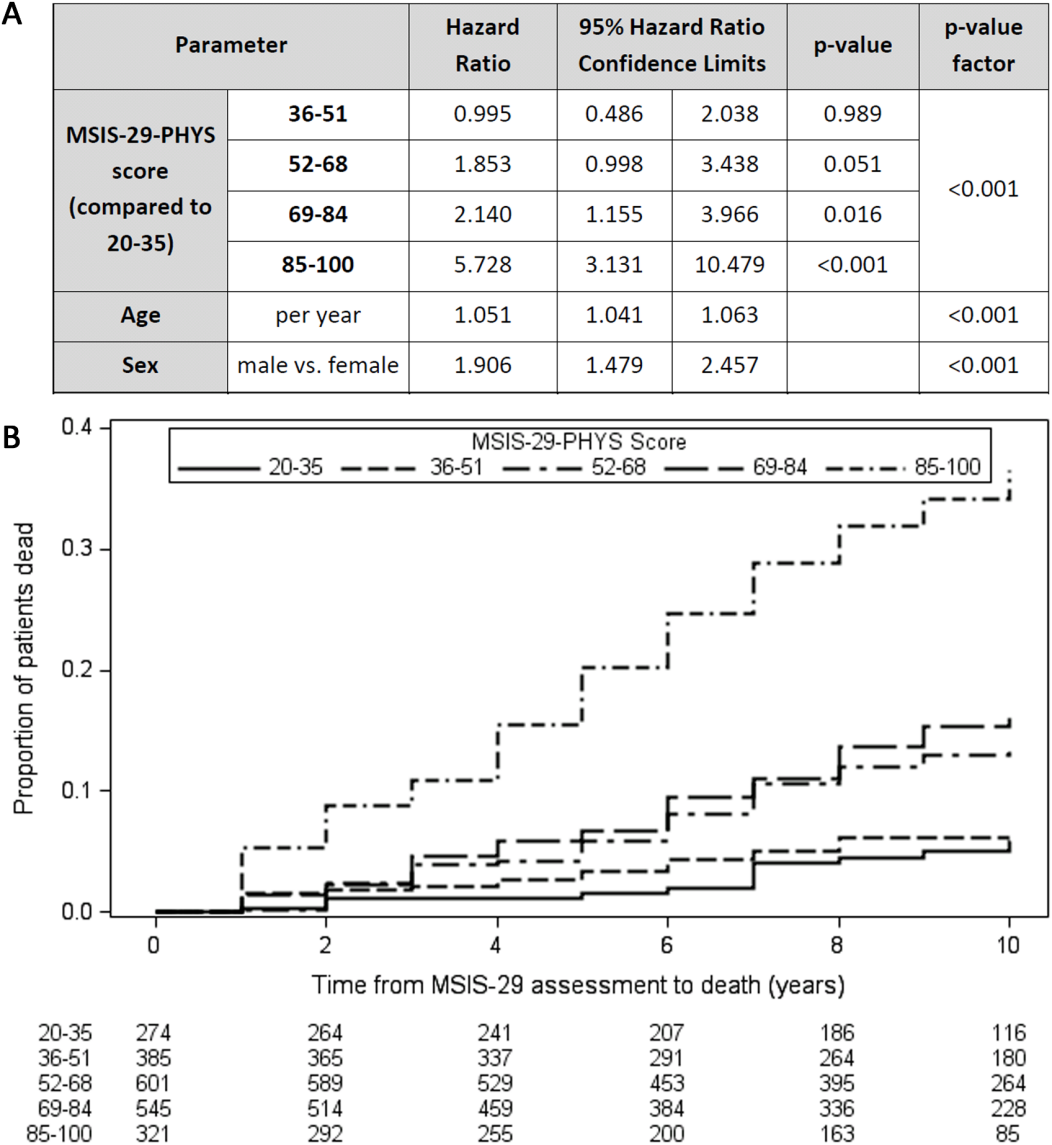
Higher MSIS-29-PHYS scores are associated with reduced survival time. (A) Table: Higher MSIS-29-PHYS score was associated with reduced survival time (greater hazard ratio for death), as were older age at first MSIS-29 completion and male sex. (B) Kaplan–Meier failure curves (n = 2,126). Note that Kaplan–Meier curves do not account for the effect of age and sex on survival time. MSIS-29, Multiple Sclerosis Impact Scale–29; MSIS-29-PHYS, MSIS-29 physical.

Cox regression models demonstrated that higher MSIS-29-PHYS score was associated with reduced survival time independently of age and sex (Wald chi-square, [degrees of freedom (df) = 4, *n* = 2,126] = 98.5, *p <* 0.001; Fig 1A). Older age at first MSIS-29 completion (Wald chi-square [df = 1, *n* = 2,126] = 88.6, *p <* 0.001) and male sex (Wald chi-square [df = 1, *n* = 2,126] = 24.8, *p <* 0.001) were also associated with reduced survival time in the model. HRs for death were greater, and reduced survival times were observed, with higher MSIS-29-PHYS score, using the ranges 20–35, 36–51, 52–68, 69–84, and 85–100 (Fig 1B). The HR for death was 5.7 in the subgroup with MSIS-29-PHYS score 85–100 compared with the subgroup with MSIS-29-PHYS score 20–35 (HR 5.7, 95% CI 3.1–10.5, *p <* 0.001). Those with higher MSIS-29-PHYS scores were more likely to be male (chi-square [df = 4, *n* = 2,126] = 25.2, *p <* 0.001) and had older age (*F*[df = 4, *n* = 2,121] = 16.1, *p <* 0.001) and longer disease duration (*F*[df = 4, *n* = 2,121] = 4.1, *p <* 0.01; Table 2).

**Table 2:** Variation in characteristics between those with different baseline MSIS-29 scores.

Characteristic	*n*	Male sex	Age	Disease duration	Baseline MSIS-29-PHYS	Baseline MSIS-29-PSYCH
**MSIS-29-PHYS score**						
20–35	274	44 (16.1%)	49.1 (11.1)	15.8 (14.0)	27.5 (4.7)	14.6 (5.0)
36–51	385	70 (18.2%)	53.0 (11.8)	17.4 (15.5)	44.0 (4.5)	19.8 (6.6)
52–68	601	146 (24.3%)	54.9 (12.0)	19.0 (15.1)	60.6 (4.8)	23.6 (7.2)
69–84	545	137 (25.1%)	55.4 (12.1)	19.5 (15.5)	76.0 (4.6)	26.7 (7.9)
85–100	321	99 (30.8%)	55.1 (11.2)	19.7 (13.2)	92.0 (4.9)	30.5 (9.0)
*p*-Value		**<0.001**	**<0.001**	**0.003**	**<0.001**	**<0.001**
**MSIS-29-PSYCH score**						
9–16	525	115 (21.9%)	55.3 (12.0)	19.8 (15.6)	46.8 (20.0)	12.9 (2.2)
17–23	598	143 (23.9%)	54.6 (12.1)	18.9 (15.0)	59.2 (17.6)	20.1 (2.0)
24–30	512	124 (24.2%)	53.6 (11.7)	17.9 (14.8)	67.2 (16.4)	26.7 (2.0)
31–37	326	83 (25.5%)	52.4 (11.8)	18.0 (14.9)	74.0 (14.7)	33.7 (2.0)
38–45	158	30 (19.0%)	51.4 (11.2)	16.2 (12.4)	82.1 (14.7)	40.8 (2.3)
*p*-Value		0.834	**<0.001**	0.053	**<0.001**	**<0.001**

Data are presented as n (percent) or mean (SD). Differences between categorical data (sex) were tested with the chi-square test. Differences between continuous data (age, disease duration, MSIS-29-PHYS, MSIS-29-PSYCH) were tested with one-way analysis of variance (ANOVA). Significant p-values are highlighted in bold.

MSIS-29, Multiple Sclerosis Impact Scale–29; MSIS-29-PHYS, MSIS-29 physical; MSIS-29-PSYCH, MSIS-29 psychological.

### A higher MSIS-29-PSYCH score is associated with reduced survival time

**Fig 2:**
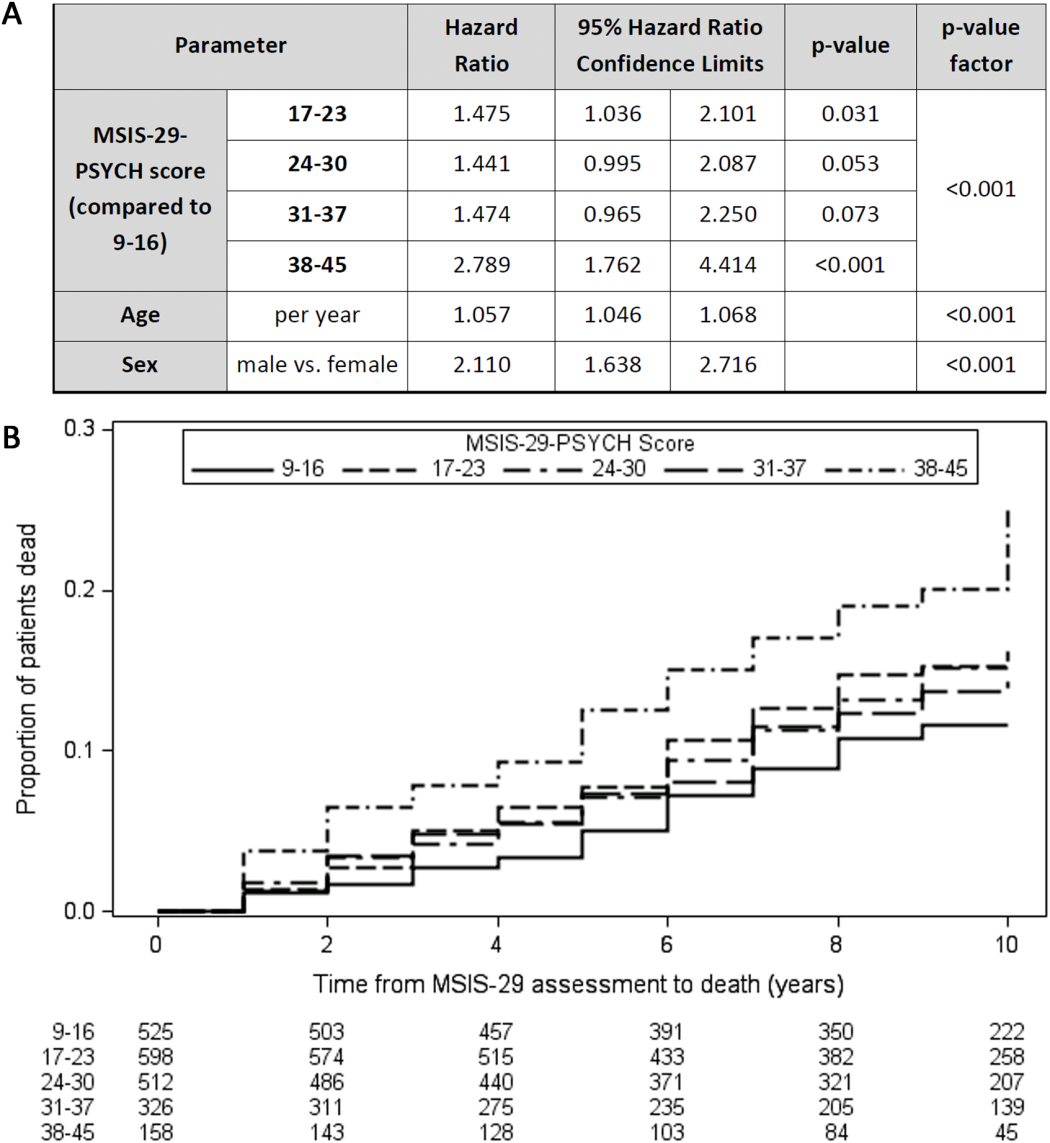
Higher MSIS-29-PSYCH scores are associated with reduced survival time. (A) Table: Higher MSIS-29-PSYCH score was associated with reduced survival time (greater hazard ratio for death), as were older age at first MSIS-29 completion and male sex. (B) Kaplan–Meier failure curves (n = 2,119). Note that Kaplan–Meier curves do not account for the effect of age and sex on survival time. MSIS-29, Multiple Sclerosis Impact Scale–29; MSIS-29-PSYCH, MSIS-29 psychological.

Similarly, Cox regression models demonstrated that higher MSIS-29-PSYCH score was associated with reduced survival time independently of age and sex (Wald chi-square [df = 4, *n* = 2,119] = 19.2, *p <* 0.001; Fig 2A), although the effect was less pronounced than with MSIS-29-PHYS. HRs for death were greater, and reduced survival times were observed, with higher MSIS-29-PSYCH score, using the ranges 9–16, 17–23, 24–30, 31–37, and 38–45 (Fig 2B). The HR for death was 2.8 in the subgroup with MSIS-29-PSYCH score 38–45 compared with the subgroup with MSIS-29-PHYS score 9–16 (HR 2.8, 95% CI 1.8–4.4, *p <* 0.001). In contrast to MSIS-29-PHYS score, higher MSIS-29-PSYCH score was not associated with male sex or longer disease duration and was associated with younger age (*F*[df = 4, *n* = 2,114] = 5.5, *p <* 0.001; Table 2).

### MSIS-29-PHYS score is correlated with prEDSS score but is independently associated with survival time

There was a strong correlation between the MSIS-29-PHYS and MSIS-29-PSYCH scores (*r*[df = 2,117] = 0.54, *p <* 0.001). There was a strong correlation between the MSIS-29-PHYS score and the prEDSS score (*r*[df = 628] = 0.52, *p <* 0.001) and a weak correlation between the MSIS-29-PSYCH score and the prEDSS score (*r*[df = 623] = 0.19, *p <* 0.001). To determine whether the MSIS-29-PHYS and MSIS-29-PSYCH scores were associated with survival time independently of prEDSS score, all measures were included in a Cox regression model, along with age and sex, in the limited number of participants who completed all 3 measures (*n* = 625; Table 3). Reduced survival time was associated with older age at baseline (per year: HR 1.07, 95% CI 1.04–1.10, *p <* 0.001), a higher prEDSS score (per 1 SD [2.2]: HR 2.0, 95% CI 1.0–3.7, *p <* 0.05), and a higher MSIS-29-PHYS score (per 1 SD [20.3]: HR 1.8, 95% CI 1.1–2.9, *p <* 0.05).

**Table 3:** Reduced survival time (greater hazard ratio for death) was associated with older age, higher prEDSS score, and higher MSIS-29-PHYS score in the limited cohort with prEDSS score available (n = 625).

Parameter	Hazard ratio	95% hazard ratio confidence limits		*p*-Value
		Lower	Upper	
prEDSS, per 1 SD (2.2)	1.952	1.025	3.718	0.042
MSIS-29-PHYS, per 1 SD (20.3)	1.762	1.057	2.938	0.030
MSIS-29-PSYCH, per 1 SD (8.7)	0.905	0.631	1.297	0.587
Age, per year	1.070	1.042	1.099	<0.001
Sex, male versus female	1.237	0.622	2.459	0.544

MSIS-29, Multiple Sclerosis Impact Scale–29; MSIS-29-PHYS, MSIS-29 physical; MSIS-29-PSYCH, MSIS-29 psychological; prEDSS, patient-reported Expanded Disability Status Scale.

### Worsening in the MSIS-29-PHYS score over 1 year is associated with reduced survival time

**Fig 3:**
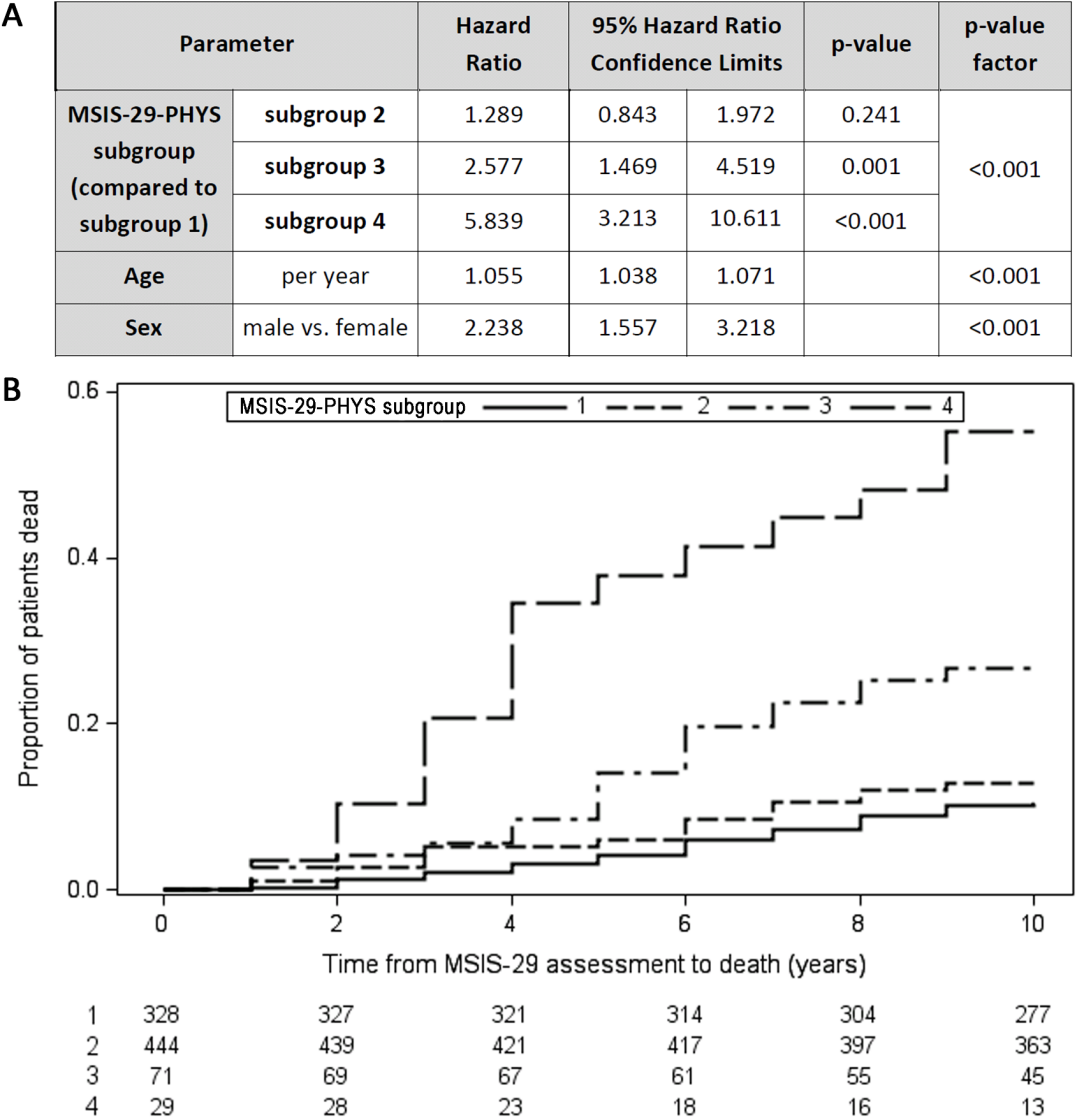
Longitudinally worsening MSIS-29-PHYS scores are associated with reduced survival time. Four subgroups are presented in this figure: Subgroup 1—initial MSIS-29-PHYS score 20–84, no worsening after 1 year (solid line); Subgroup 2—initial MSIS-29-PHYS score 20–84, worsening after 1 year (short-dashed line); Subgroup 3—initial MSIS-29-PHYS score 85–100, no worsening after 1 year (dot-dashed line); Subgroup 4—initial MSIS-29-PHYS score 85–100, worsening after 1 year (long-dashed line). (A) Table: Subgroups 1 and 2 had no statistically significant difference in survival time (HR = 1.289, 95% CI 0.843–1.972, p = 0.241). Subgroup 4 had reduced survival time compared with subgroup 3 (HR = 2.266, 95% CI 1.163–4.413, p = 0.016). p < 0.001 for differences between the 4 subgroups. (B) Kaplan–Meier failure curves (n = 872). Note that Kaplan–Meier curves do not account for the effect of age and sex on survival time. HR, hazard ratio; MSIS-29, Multiple Sclerosis Impact Scale–29; MSIS-29-PHYS, MSIS-29 physical.

The MSIS-29 questionnaire was repeated after 1 year in a subgroup (*n* = 872) of those who had originally completed the MSIS-29 in 2004. Comparing MSIS-29-PHYS stable/improving score participants with MSIS-29-PHYS worsening score participants (change in MSIS-29-PHYS ≤ 0 versus > 0), there was no statistically significant difference in mortality (Cox regression adjusted for age and sex: *n* = 872, HR = 1.2, 95% CI 0.9–1.2, *p* = 0.28). However, in the subgroup of participants whose initial MSIS-29-PHYS score was 85–100, a longitudinal worsening of MSIS-29-PHYS score was associated with reduced survival time (HR = 2.3, 95% CI 1.2–4.4, *p* = 0.016; Fig 3). This was not apparent in the subgroup with initial MSIS-29-PHYS score 20–84 (HR = 1.3, 95% CI 0.8–2.0, *p* = 0.24; Fig 3). Fig 3B shows the survival curves for the 4 subgroups.

## Discussion

This study reports that higher MSIS-29 score is associated with reduced survival time in a large observational cohort of people with MS. MSIS-29-PHYS and MSIS-29-PSYCH scores were both associated with survival time, although MSIS-29-PHYS score has stronger prognostic value, and associates with survival time independently of age, sex, and prEDSS score in a Cox regression model. In addition, in the subgroup with initial MSIS-29-PHYS score 85–100, a 1-year longitudinal worsening of MSIS-29-PHYS score is associated with an even worse prognosis. This finding shows that how a person with MS answers these questions is important and relates to how well they may do in terms of health in the future. To our knowledge, this is the first study to associate PROs with survival outcomes in any neurological disease.

This study benefitted from the MSSTB cohort in several ways. We believe this is the largest cohort with reported MSIS-29 results to date, other than 1 web-based cohort [28,29], and the MSSTB has by far the longest follow-up after MSIS-29 completion (up to 10 years, median 9 years). This cohort had 264 deaths within the study period. This number of deaths allowed the prognostic value of MSIS-29 scores for survival time to be studied.

One should consider the external validity of this cohort’s results to the general MS population. The MSSTB recruitment strategy is based entirely in the community, relying upon community-based presentations and a quarterly magazine, *MS Matters*, distributed to approximately 30,000 members of the Multiple Sclerosis Society of Great Britain and Northern Ireland [30]. Moreover, the MSSTB population has previously been shown to be representative of the UK MS population in terms of disease characteristics and clinical milestones over the course of the disease [23]. The spread of MSIS-29 scores in this study was comparable with that reported in other large cohort studies [28,29]. In addition, factors that are known to associate with reduced survival, such as male sex, older age at baseline, and higher prEDSS score, were also found to associate with reduced survival time in this cohort, along with MSIS-29 score [31–33]. However, at the time of enrolment into the MSSTB, participants are often late in their disease course, as evidenced by this study’s mean disease duration of 18.5 years and mean prEDSS score of 5.7 at baseline MSIS-29 questionnaire. Therefore, this study likely underrepresents those with earlier disease and less disability, and it is uncertain how MSIS-29 scores and their prognostic value for mortality will vary in this group. One might hypothesize that those with earlier disease would have lower MSIS-29 scores and increased survival times, on average [29].

There are several other limitations to this study. Only a limited cohort completed the prEDSS questionnaire (*n* = 630), and only a limited cohort completed a longitudinal MSIS-29 (*n* = 872), mostly because of changes to the study protocol over time. Also, this study used a prEDSS rather than the traditional physician-reported EDSS, although these have previously been shown to correlate well [24]. Data on disease subtype, relapses, and disease-modifying therapy were not available for this study, and could influence the relationship between MSIS-29 score and survival time. Like most clinical outcome measures, PROs are susceptible to random measurement error, and hence regression dilution bias likely causes a decrease in the prognostic value of MSIS-29 for mortality [34]. However, previous studies have reported the test–retest reliability of MSIS-29-PHYS and MSIS-29-PSYCH to be high (intraclass correlation coefficients of 0.94 and 0.87, respectively), and so this effect is likely minimal [14].

In MS research, PROs such as MSIS-29 offer several advantages over traditional physician-assessed outcome measures such as the EDSS [2]. Interrater EDSS variability is high [17,35]. The EDSS also has a limited sensitivity to change over the 2- to 3-year time scale of clinical trials [36,37]. This is especially true in progressive MS, where the EDSS often does not capture changes in arm function or subtle changes in mobility [38]. MSIS-29 can be more responsive to clinically relevant change over short time frames [15,39]. PROs including MSIS-29 can also be sent to large cohorts of people with MS, and completed by post or online [29]. PROs could therefore enable large cohort studies, which would otherwise be financially unfeasible, such as comparative clinical effectiveness research. PROs validated against hard clinical endpoints could also be incorporated into patient registries to help address key questions in personalised medicine relating to prognosis, predicting response to treatment, and assessing response to treatment. These are increasingly important issues in MS and other neurological diseases like epilepsy, stroke, Parkinson disease, and spasticity, where a range of therapies are now available but questions remain regarding how to treat the individual.

Outside of the research setting, PROs can benefit individual patients directly if they are utilized in routine clinical practice. Oncology has again led the way, where PROs are routinely used to enhance patient care [5]. PROs can help screen for changes in physical or psychological symptoms, and identify unmet health, care, and support needs. They can be used as a decision aid when devising or evaluating treatment plans [40]. Patients and doctors can have differing views on which outcomes matter most, and the effective use of PROs can help refocus care goals to the views of the individual patient [41,42]. This might also empower patients towards improved self-management of their condition [43]. When assessed in a randomised controlled trial, PROs enhanced doctor–patient communication and improved patient health-related quality of life and emotional well-being [4].

On a healthcare provision level, PROs can also be used to audit quality of care within a service or to compare quality of care between services [44]. PROs are often now incorporated into automated electronic systems for data collection, with high user compliance [45,46]. As well as providing direct benefit to patients, these systems can also feed into patient registries to help address research questions.

Further research questions emerge from this study. With this study having shown that MSIS-29 score is associated with death, similar methods could be used to investigate whether MSIS-29 score is associated with disability outcomes, such as time until wheelchair use. The effect of disease-modifying treatment on MSIS-29 and long-term clinical endpoints needs further attention, to assess whether MSIS-29 scores could be used as a surrogate for response to treatment. Multiple variables, including other PROs collected at multiple time points, could be incorporated into more complex models to better predict outcomes in large cohorts.

PROs will continue to be used in interventional studies, in part to satisfy labelling and promotional claims in post-licensing marketing [1,7]. This study argues that the MSIS-29 questionnaire can offer more than this, since its association with hard clinical endpoints supports its use as a meaningful clinical outcome to inform care decision-making. In oncology, PROs are now established as influential and clinically relevant measures, and it is accepted that the classic clinical endpoints do not fully capture the benefits, risks, and costs of treatment [8–10]. MS and neurology research will continue to rely upon clinical trials, as well as ‘big data’ gathered from clinical registries. The careful incorporation of PROs can enrich such datasets and allow the investigation of research questions beyond what traditional physician-based assessments can offer.

## Supporting information

S1 Appendix Description of how to request access to the MSIS-29 questionnaire and prEDSS questionnaire, and a copy of the MSIS-29 questionnaire. (DOCX)

S2 Appendix Description of the analysis plan, and historical changes made to the study. (DOCX)

S3 Appendix Imperial College London medical student BSc project outlines 2013–14, with a description of MSIS-29 project. (PDF)

## Abbreviations

95% CI: 95% confidence interval
df: degrees of freedom
EDSS: Expanded Disability Status Scale
HR: hazard ratio
MS: multiple sclerosis
MSIS-29: Multiple Sclerosis Impact Scale–29
MSIS-29-PHYS: MSIS-29 physical
MSIS-29-PSYCH: MSIS-29 psychological
MSSTB: MS Society Tissue Bank
prEDSS: patient-reported Expanded Disability Status Scale
PRO: patient-reported outcome
SD: standard deviation
