# Supplementary material for: Patient-reported outcomes and survival in multiple sclerosis: A 10-year retrospective cohort study using the Multiple Sclerosis Impact Scale–29
Source: PLoS Med. 2017 Jul 10;14(7):e1002346. doi: 10.1371/journal.pmed.1002346 (PMC5503162; doi:10.1371/journal.pmed.1002346)
Supplement: S1 Appendix — (DOCX) [file pmed.1002346.s001.docx]

**Supplementary Appendix 1** - Statement on the availability of the MSIS-29 and prEDSS questionnaires

- The MSIS -29 is copy-right by the Neurological Outcome Measures Unit. Permission to use the scale can be obtained by contacting Dr. Jeremy Hobart at J.Hobart@ion.ucl.ac.uk
- The prEDSS is copy-right by L. Kappos, Department of Neurology, University Hospitals Basel. Permission to use the scale can be obtained by contacting Dr. Ludwig Kappos at lkappos@uhbs.ch
- A copy of the MSIS-29 can be viewed online at the following URLs:

[www.biomedcentral.com/content/supplementary/1471-2377-8-2-s1.doc](http://www.biomedcentral.com/content/supplementary/1471-2377-8-2-s1.doc) (accessed April 2017).

- A copy of the prEDSS is available on contacting Dr. Ludwig Kappos at lkappos@uhbs.ch
