# Supplementary material for: Patient-reported outcomes and survival in multiple sclerosis: A 10-year retrospective cohort study using the Multiple Sclerosis Impact Scale–29
Source: PLoS Med. 2017 Jul 10;14(7):e1002346. doi: 10.1371/journal.pmed.1002346 (PMC5503162; doi:10.1371/journal.pmed.1002346)
Supplement: S2 Appendix — (DOCX) [file pmed.1002346.s002.docx]

**Supplementary Appendix 2**

**Analysis history for the study described in: Raffel J, Wallace A, Gveric D, Reynolds R, Friede T, Nicholas R. Patient-reported outcomes and survival in multiple sclerosis: a 10-year retrospective cohort study using the MSIS-29. PLOS Medicine.**

We did not pre-register or publish a detailed analysis plan for this study. The analysis of data is described in the study methodology. Details on the history of this study are given below:

1. The MSIS-29 questionnaire was added to the standard protocol for the MS Society Tissue Bank (MSSTB) in 2003/4 “because it is a patient-based outcome that provides a good clinical picture of a person’s MS” (MSSTB Annual Report – March 2003 to February 2004; section 6, p9). This was implemented by two of the study authors (RR, RN), to allow the correlation of “clinical information on donors during life” with data collected by the MSSTB at death (MSSTB Annual Report – March 2003 to February 2004; section 1, p1). Raw data were accrued from 2004-2014, including questionnaire and mortality data, and stored as part of standard tissue bank practice.
2. The study was motivated by the primary research question “Can MSIS-29, or change in MSIS-29, be prognostic for mortality data in multiple sclerosis?” This study question was defined prior to the analysis of any data. A project proposal was advertised in 2013, prior to the collation or analysis of any data, for a student to investigate the primary research question of whether baseline MSIS-29 score, or change in MSIS-29 score, was associated with death (S3 Appendix). In this short proposal, the statistical plan was described only briefly as “mean score [MSIS-29] relationship to disease length and time to outcome [death]. Change in score [MSIS-29] and relationship to outcome [death]' (explanatory notes in parentheses).
3. Data collation took place from 2014 of all data presented in this manuscript, including MSIS-29, prEDSS, mortality, and demographic data. Imputation rules were decided upon at this point, and have not changed. A preliminary univariate multivariable regression analysis concluded that MSIS-29 scores was associated with survival.
4. Methods for statistical analysis were refined in 2016 after consultation with a statistician (author TF), to develop the Cox proportional hazard models presented in this manuscript. This was done to improve analysis methods to those suited to the dataset from a statistical perspective, and had no impact on the relationships and overall message reported in the results. Subgroup analyses were also decided upon at this point, including the categorisation of participants into five subgroups based upon baseline MSIS-29 scores. The subgroup range values were originally chosen after interrogation of the data to achieve a compromise between ‘number of participants’ and ‘number of deaths’ in each subgroup, to allow for sufficient power in statistical analyses.
5. After peer-review from PLOS Medicine, the subgroup range values for MSIS-29 groups were modified so that the five subgroups were equally spaced in terms of MSIS-29 scores. This change was made to make the subgroup ranges for MSIS-29 subgroups more intuitive to the reader. This had no impact on the relationships and overall message reported in the results. Other new analyses performed after PLOS Medicine peer review were the comparison of subgroups shown in Table 1, and the comparison of subgroups by MSIS-29 score shown in Table 2.
