# Supplementary material for: Patient-reported outcomes and survival in multiple sclerosis: A 10-year retrospective cohort study using the Multiple Sclerosis Impact Scale–29
Source: PLoS Med. 2017 Jul 10;14(7):e1002346. doi: 10.1371/journal.pmed.1002346 (PMC5503162; doi:10.1371/journal.pmed.1002346)
Supplement: S3 Appendix — (PDF) [file pmed.1002346.s003.pdf]

BSc Neuroscience & Mental Health

-

Student Project Outlines 2013-14

(Amended 24-11-13)

PROJECT 47

CLINICAL PROJECT

Academic Supervisor:
Dr Richard Nicholas (r.nicholas@imperial.ac.uk)

Project Title: Relationship of patient reported outcome to clinical outcomes in Multiple Sclerosis

Number of students required: 1

Background to Project: The UK MS tissue bank collects tissue for research. Subjects register when alive and complete patient reported outcome assessments at entry. Some years ago we performed an outcome assessment on subjects twice one year apart. We have also collected data when everyone has registered together with other details of their condition. We wish to enter this data and analyse whether it predicts clinical outcome.

Research question or hypothesis student will investigate: Does change in MSIS-29 predict death, does entry MSIS-29 predict outcome and baseline perceived state

Rationale for research plan: We need to understand the utility of patient reported outcomes link to clinical outcome measures so that they can be used in trials as valid outcome measures.

Sample and methods (techniques) student will use: Enter data on spreadsheet and then run a statistical analysis.

Proposed scheme of analysis: Mean score relationship to disease length and time to outcome. Change in score and relationship to outcome

Will the research involve?

- Work with Patients (non-invasive, non-interventional) No
- Access to confidential patient information Yes
- Handling of Human blood, serum or unfixed tissue No
- Deliberate work with a Group 2 or 3 Human Pathogen No
- Deliberate work with a Class 2 or higher Genetically Modified Organism No

Ethical approval obtained: 08/MREC/09/31+5

PROJECT 48

LABORATORY PROJECT

Academic Supervisor:
Prof Steve Gentleman (s.gentleman@imperial.ac.uk)
Dr Ronald Pearce (ronald.pearce@imperial.ac.uk)

Project Title: Differential expression of galanin in the basal forebrain in Parkinson's disease and Parkinson's disease dementia

Number of students required: 1

Background to Project: Parkinson's disease (PD) is primarily a movement disorder but up to 80% of patients with a longer course of disease will develop significant cognitive impairment leading to
